# Diethylcarbamazine Increases Activation of Voltage-Activated Potassium (SLO-1) Currents in *Ascaris suum* and Potentiates Effects of Emodepside

**DOI:** 10.1371/journal.pntd.0003276

**Published:** 2014-11-20

**Authors:** Samuel K. Buxton, Alan P. Robertson, Richard J. Martin

**Affiliations:** Department of Biomedical Sciences, Iowa State University, Ames, Iowa, United States of America; McGill University, Canada

## Abstract

Diethylcarbamazine is a drug that is used for the treatment of filariasis in humans and animals; it also has effects on intestinal nematodes, but its mechanism of action remains unclear. Emodepside is a resistance-busting anthelmintic approved for treating intestinal parasitic nematodes in animals. The novel mode of action and resistance-breaking properties of emodepside has led to its use against intestinal nematodes of animals, and as a candidate drug for treating filarial parasites. We have previously demonstrated effects of emodepside on SLO-1 K^+^-like currents in *Ascaris suum*. Here, we demonstrate that diethylcarbamazine, which has been proposed to work through host mediated effects, has direct effects on a nematode parasite, *Ascaris suum*. It increases activation of SLO-1 K^+^ currents and potentiates effects of emodepside. Our results suggest consideration of the combination of emodepside and diethylcarbamazine for therapy, which is predicted to be synergistic. The mode of action of diethylcarbamazine may involve effects on parasite signaling pathways (including nitric oxide) as well as effects mediated by host inflammatory mediators.

## Author Summary

Filarial parasites and soil-transmitted nematodes (STNs) are Neglected Tropical Diseases (NTDs) that affect millions of people in the developing world. There is an urgent need for novel drugs and improved use of existing drugs, because of concerns about the development of resistance. The mode of action of one of these drugs, diethylcarbamazine, remains unclear, despite the fact that it has been used for a long time for treatment and prevention of filariae and STNs. The resistance-busting anthelmintic emodepside also has effects against filariae and STNs, with a mode of action that involves activation of nematode SLO-1 K^+^ channels. The effects of both diethylcarbamazine and emodepside may be increased by inflammatory mediators, which suggests that the effects of diethylcarbamazine and emodepside will be additive. We used our *Ascaris suum* preparation to test the activation of SLO-1 K^+^ channels by diethylcarbamazine and its potentiating effect on emodepside. Our results suggest potential for diethylcarbamazine and emodepside in combination therapy for parasitic nematodes.

## Introduction

Infections with parasitic nematodes are a global concern for human and animal health. These infections come in the form of gastrointestinal nematodes, like ascariasis infections, hookworm infections and trichuriasis infections, as well as infections transmitted by biting insects, like filariasis. Over 1 billion people are infected with parasitic nematodes [1], especially in the tropical regions where the combination of poor sanitation, warm and moist conditions creates the conducive environment for survival and spread of these parasites. Infections of parasitic nematodes of farm animals cause massive loss of food production and also lead to animal welfare issues. Parasitic nematode infections cause cognitive impairment of humans and, in humans and animals, stunted growth, anemia, sometimes swollen limbs and sometimes death. In the absence of effective vaccines or sanitation, anthelmintic drugs are required for both treatment and prophylaxis. There are a limited number of drug classes available and their frequent use has produced resistance in animals [2] and concerns about development of resistance in humans [3], [4]. One way to slow the speed of development of resistance is to use the drugs that are available in a more targeted manner [5] and to use synergistic combinations of drugs [6]. In this paper we explore effects of diethylcarbamazine and describe the interactive effects of the combination of emodepside and diethylcarbamazine.

Emodepside, a semisynthetic derivative of PF1022A, has a novel mechanism of action, different from other anthelmintics; it is effective against a broad spectrum of parasitic nematodes, including soil-transmitted nematodes [7], [8]. Emodepside has a complex mode of action involving activation of a voltage-activated calcium-dependent K^+^ channel (SLO-1) at the neuromuscular junction [9], [10] and potentiation of its effects by drugs that increase levels of nitric oxide [11]. Emodepside has potential as a drug for the treatment of filarial parasites: it is larvicidal and adulticidal *in vitro* and *in vivo* but the efficacy of emodepside against filariae depends on species, being quite low against *Brugia pahangi* and *Brugia malayi* in comparison to other filariae [12], [13], [14].

Diethylcarbamazine citrate is an established antifilarial drug which has been used since 1947 for the treatment of lymphatic filariasis and loiasis. It is still an important and effective antifilarial drug but its mode of action is not fully described. Diethylcarbamazine has been suggested to have an indirect, host mediated mode of action: it appears to alter host arachidonic acid and nitric oxide metabolic pathways, which in an unknown way leads to immobilization and sequestration of the microfilariae [15]. Diethylcarbamazine activity against *B. malayi* microfilariae is abolished in inducible nitric oxide synthase knockout mice (iNOS^−/−^), suggesting that diethylcarbamazine activity is dependent on host inducible nitric oxide synthase (iNOS) and nitric oxide, [16].

We were interested to determine: how diethylcarbamazine would affect calcium-dependent SLO-1 K^+^ currents in isolated *Ascaris suum* muscle flap preparations and; how diethylcarbamazine interacts with emodepside. The interest was prompted by observations in vertebrates [17] which show that nitric oxide activates SLO-1 K^+^ channels and observations on *Ascaris* indicating the presence nitric oxide synthase [18] and of SLO-1 K^+^ channels which show positive modulation by a nitric oxide pathway [11], [19]. We hypothesized that diethylcarbamazine, with effects on arachidonic acid and nitric oxide pathways, may increase activation of SLO-1 K^+^ currents in *Ascaris suum* muscle and potentiate effects of emodepside on membrane potential. We conducted experiments in the presence of sufficient calcium to allow activation of the SLO-1 K^+^ currents. Here we show that diethylcarbamazine, by itself, can increase activation of SLO-1 K^+^ currents and potentiate effects of emodepside.

## Methods

Adult *Ascaris suum* were collected weekly from JBS Swift and Co. pork processing plant, Marshalltown, IA and maintained for up to 4 days in Locke's solution (NaCl 155 mM, KCl 5 mM, CaCl_2_ 2 mM, NaHCO_3_ 1.5 mM, glucose 5 mM) at 32°C. About 1 cm of the anterior part of the worm, 4 cm from the head, was cut-out and the cylindrical worm piece cut open along a lateral line to form a muscle flap. After removing the gut to expose muscle cells, the muscle flap was pinned to a 35×10 mm Sylgard-lined Petri-dish containing low-potassium, high-calcium *Ascaris* perienteric fluid (APF) (mM: NaCl 23, Na acetate 110, KCl 3, CaCl_2_ 6, MgCl_2_ 5, glucose 11, HEPES 5, pH adjusted to 7.6 with NaOH). A 20-gauge perfusion needle, placed directly over the muscle bag being recorded from, delivered the drugs in APF at a rate of 4 mL min^−1^. We employed the two-micropipette current-clamp and voltage-clamp techniques to investigate the effects of diethylcarbamazine and emodepside on *A. suum* muscle bag. Micropipettes were pulled on a Flaming Brown Micropipette Puller (Sutter Instrument Co., Novato, CA, USA) and filled with 3 M potassium acetate. Resistance of the voltage-sensing micropipettes was between 20–30 MΩ but the tip of the current-injecting micropipette was broken to have a resistance of 3–6 MΩ. A 1320A Digidata, an Axoclamp 2B amplifier and pClamp 8.2 software (Molecular Devices, Sunnyvale, CA, USA) were used for the recordings. The resting membrane potential of cells selected for recording were stable and between −25 mV and −35 mV and had input conductances less than 4.0 µS. The current-clamp protocol consisted of injection of 40 nA hyperpolarizing pulses for 500 ms and recording the change in membrane potential with the voltage-sensing micropipette. In the voltage-clamp protocol, the muscle bag was held at −35 mV and then stepped to 0, 5, 10, 15, 20, 25 and 30 mV to activate the K^+^ currents. We used a leak subtraction protocol [11] that averaged four 5 mV hyperpolarizing pre-pulses to obtain the leak subtraction current before each depolarizing step and which was scaled by the amplitude of the depolarizing step for leak subtraction. The leak subtraction was under the control of pClamp software. The leak subtraction procedure was not modified otherwise by voltage, emodepside or emodepside and diethylcarbamazine. Recordings were rejected if the conductance of the muscle cells increased abruptly, indicating cell membrane damage, or if the conductance increased above 4 µS. Acquired data were displayed on a Pentium IV desktop computer and the currents were leak-subtracted. We analyzed the leak subtracted K^+^ current at the 0 mV step potential because the emodepside effect was biggest at this potential [11]. All chemicals and drugs were purchased from Sigma Aldrich (St Louis, MO, USA) except emodepside, which was generously supplied by Achim Harder (Bayer HealthCare AG, Leverkusen, Germany). Emodepside stocks of 2 mM in 100% DMSO were prepared every two weeks. The working emodepside concentration was prepared so that the final DMSO concentration did not exceed 0.1%. To avoid problems with emodepside coming out of solution, we did not keep it longer than the two weeks and in some cases, we prepared fresh emodepside for every experiment. Effects of drug applications were measured after 10 min and post-drug measurements made after a 20 min wash in drug-free solutions. Graph Pad Prism Software (version 5.0, San Diego, CA, USA) and Clampfit 9.2 (Molecular Devices) were used for data analysis. The activation curve was fitted by the Boltzmann equation *G*  =  *G*_max_/[1 + exp {(*V*_50_ - *V*)/*K*_slope_}], where *G* =  conductance, *G*max  =  maximal conductance change, *V_50_*  =  half-maximal voltage and *K*_slope_  =  slope factor.

## Results

### Diethylcarbamazine increases voltage-activated SLO-1 currents and hyperpolarizes V_50_

**Figure 1:**
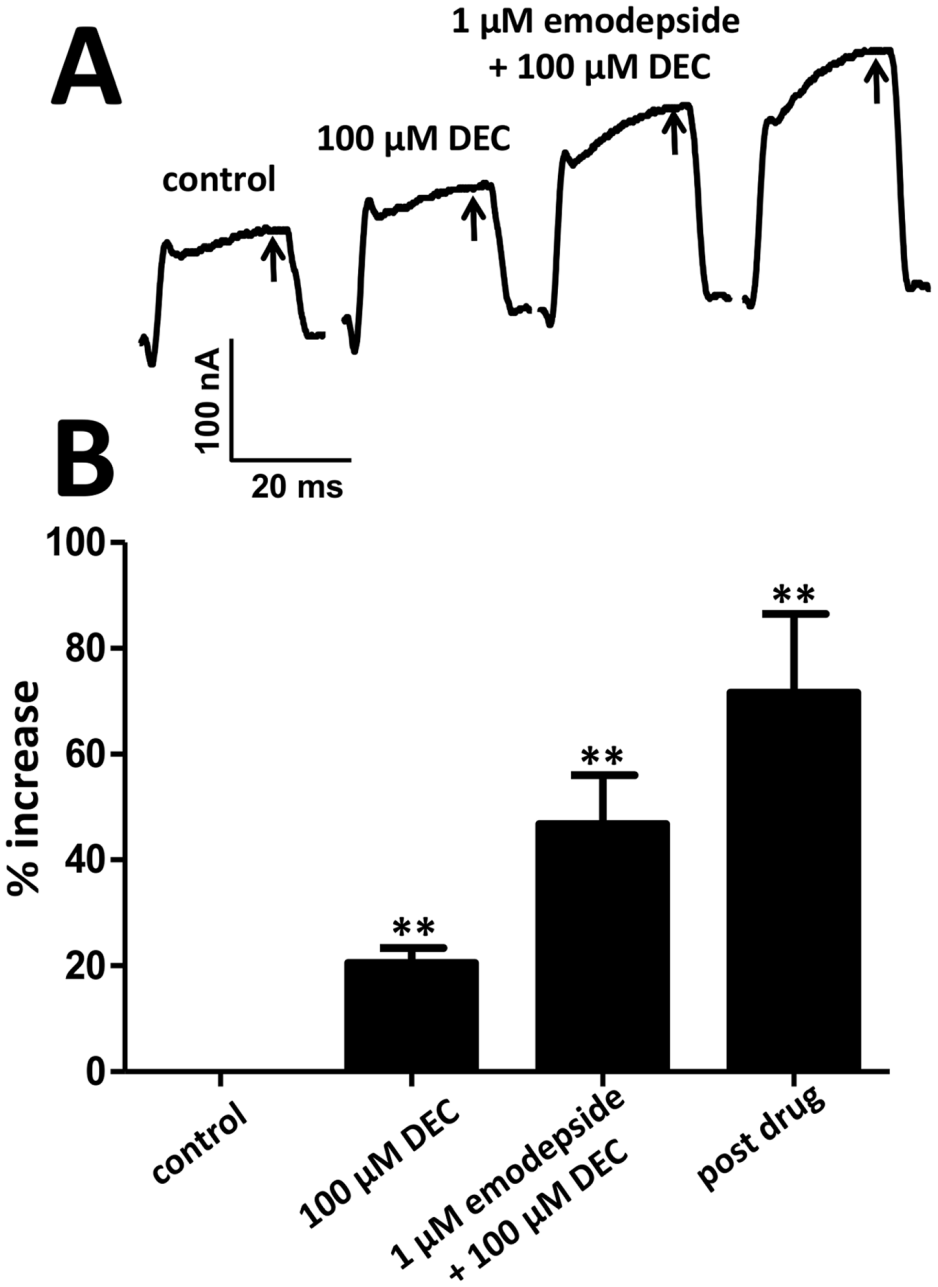
Effect of emodepside and diethylcarbamazine on SLO-1 K^+^ currents. **A**: Representative leak-subtracted K^+^ current traces of the control current, 100 µM diethylcarbamazine effect, 1 µM emodepside plus 100 µM diethylcarbamazine effect and the post-emodepside current at the 0 mV step potential. The post drug currents were measured after 20 min wash in drug-free solution. **B**: Bar chart (mean ± SEM) of % increase in the K^+^ currents at the step potential of 0 mV produced by 100 µM diethylcarbamazine (p<0.01, n = 4, *paired t-test*), 1 µM emodepside plus 100 µM diethylcarbamazine (p<0.01, n = 4, *paired t-test*) and at the post-drug (emodepside) 20-min period (p<0.01, n = 4, *paired t-test*).

We have shown that emodepside increases SLO-1 K^+^ currents by shifting *V_50_* of the voltage-activation curve in the hyperpolarizing direction [11]. Consequently, we investigated the effects of diethylcarbamazine on the voltage-activation of SLO-1 K^+^ currents by itself and in combination with emodepside. Fig. 1 A shows a representative recording of the control SLO-1 K^+^ current produced by holding the cell at -50 mV and stepping to 0 mV; the arrow, Fig. 1 A indicates the time-point in the depolarizing pulse at which the current measurements were taken for plotting results. The application of diethylcarbamazine by itself significantly increased the peak K^+^ currents by 21±3% (p<0.01, n = 4, paired t-test, Fig. 1 A & B). The application of 100 µM diethylcarbamazine and 1 µM emodepside together increased the SLO-1 K^+^ currents by 47±9%, (p<0.01, n = 4, paired t-test, Fig. 1 A & B). The K^+^ currents were further increased over 20 min despite wash out by 72±15% (p<0.01, n = 4, paired t-test), Fig. 1 A & B. We have previously described how effects of emodepside continue to increase slowly over time and do not wash off [11]. Similar effects were observed in this set of experiments during the post-emodepside and post-diethylcarbamazine period. We therefore measure drug effects after a fixed period of 10 min following drug applications to standardize observations [11]. The slow increase and lack of wash-off may be explained by the lipophilic nature of emodepside and activation of signaling cascades that include nitric oxide.

**Figure 2:**
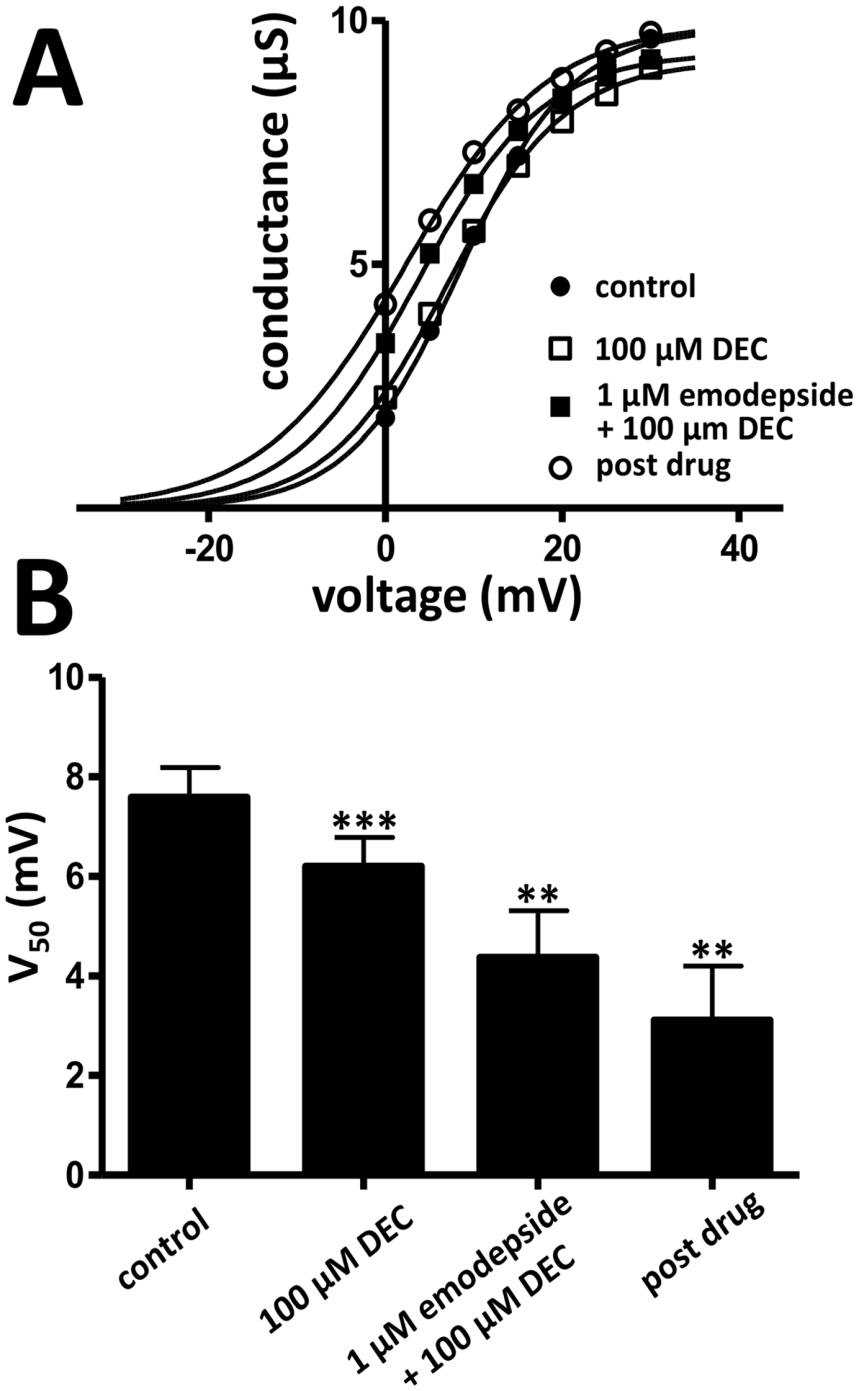
Effects of diethylcarbamazine and diethylcarbamazine combination on the activation curve of the SLO-1 K^+^ current. **A**: Sample activation curve of the K^+^ currents. The conductance-voltage plot was fitted to the Boltzmann equation. Diethylcarbamazine shifted the *V_50_* in the hyperpolarizing direction and the shift was sustained during 1 µM emodepside plus 100 µM diethylcarbamazine and the post-emodepside periods. Control (•) *V_50_* = 8.5 mV; 100 µM diethylcarbamazine (□) *V_50_* = 7.0 mV; 1 µM emodepside +100 µM diethylcarbamazine (○) *V_50_* = 3.7 mV; post-emodepside (▪) *V_50_* = 2.0 mV. **B**: Bar chart (mean ± s.e.) of the change in *V_50_* of the K+ currents produced by diethylcarbamazine and emodepside plus diethylcarbamazine (**p<0.01, ***p<0.001, n = 4–5, *paired t-test*).

Next, we determined the effect of emodepside plus diethylcarbamazine on the voltage-sensitivity of the SLO-1 K^+^ current, *V_50_*. In the representative conductance-voltage plot displayed in Fig. 2 A, diethylcarbamazine by itself shifted the curve in the hyperpolarizing direction, making the channels more sensitive to depolarization. In the presence of both emodepside and diethylcarbamazine, there was a further hyperpolarizing shift of *V_50_*. Fig. 2 B shows that 100 µM diethylcarbamazine decreased the average *V_50_* from 7.6±0.6 mV to 6.2±0.6 mV (p<0.001, n = 5, *paired t-test*, Table 1). In the presence of the combination of 100 µM diethylcarbamazine and 1 µM emodepside, *V_50_* decreased to 4.4±0.9 mV (p<0.01, n = 5, *paired t-test*, Table 1) and the decrease continued to 3.1±1.1 mV during the post-emodepside period (p<0.01, n = 4, *paired t-test*, Table 1). In separate experiments we tested the effects of 1 µM emodepside alone and observed that by itself only produced a 30% increase the SLO-1 K^+^ current (Table 1). These experiments demonstrated that diethylcarbamazine mimicked the effect and, increased the effect of emodepside on *V_50_* of the SLO-1 K^+^.

**Table 1:** Summary of diethylcarbamazine and emodepside effects on membrane potential and SLO-1 K^+^ currents.

Effects	n	emodepside (1 µM)^a^	n	emodepside (10 µM)^a^	n	diethylcarbamazine (100 µM)	n	emodepside (1 µM) + diethylcarbamazine (100 µM) combination
**Membrane potential (mV)**	10	−5.1±0.75	6	−7.7±1.2	5	−1.2 ±1.0	5	−10.0±2.0
**% control SLO-1 K^+^ currents (nA) at step 0 mV**	9	130±4	6	130±10	4	121±3	4	147±9
***V_50_*** ** (mV)**		NR	6	2.9±0.3	4	1.4±0.6	4	3.2±0.6

The effects of emodepside (1 µM, 10 µM), diethylcarbamazine (100 µM) and emodepside (1 µM) + diethylcarbamazine (100 µM) on membrane potential, on SLO-1 K^+^ at the step potential of 0 mV and on the *V_50_* of the activation curve.

a From reference [11]. NR  =  Small, Not Resolvable.

### Effects of diethylcarbamazine on membrane potential and on the hyperpolarizing effect of emodepside

**Figure 3:**
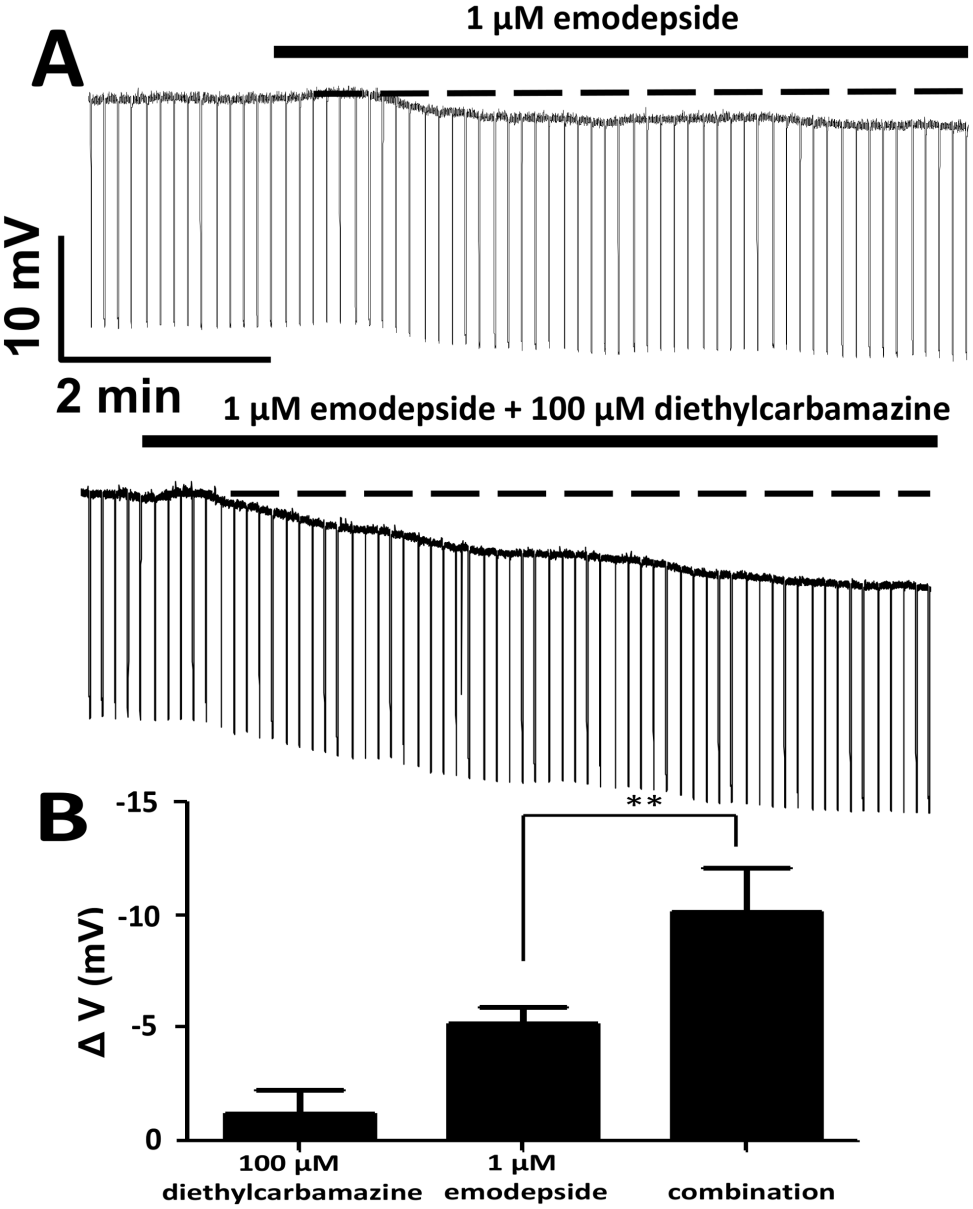
Effect of emodepside and diethylcarbamazine on *A. suum* membrane potential. **A**: Representative current-clamp traces showing the membrane potential before, during 100 µM diethylcarbamazine application (top trace) and during application of 1 µM emodepside plus 100 µM diethylcarbamazine (lower trace). Note there is no change in the input conductance, represented by the thickness of the trace. The time delay between the end of the application of 100 µM diethylcarbamazine and 1 µM emodepside plus 100 µM diethylcarbamazine was 10 minutes. The change in membrane potential between the beginning of the 100 µM diethylcarbamazine and application of 1 µM emodepside plus 100 µM diethylcarbamazine was a hyperpolarization of 1.5 mV. **B**: Bar chart (mean ± SEM) of diethylcarbamazine effect on emodepside-induced hyperpolarization. 100 µM diethylcarbamazine increased the hyperpolarization caused by 1 µM emodepside (p<0.01, n = 5, *unpaired t-test*).

Although 10 µM diethylcarbamazine was without effect on the resting membrane potential, 100 µM diethylcarbamazine produced a small, slow ∼1 mV hyperpolarization of the membrane potential in 3 of 5 separate preparations. However the effect of 100 µM diethylcarbamazine after emodepside pre-treatment was much bigger. Fig. 3A shows a representative recording where 1 µM emodepside caused a hyperpolarization of −5.3 V and in the presence of the 1 µM emodepside, 100 µM diethylcarbamazine caused a hyperpolarization of −11.1 mV.

In a series of experiments on ten different preparations, 1 µM emodepside by itself, caused a slow hyperpolarization of −5.1±0.8 mV of the *Ascaris suum* muscle membrane potential (p<0.01, n = 10, *paired t-test,* Fig. 3B). Addition of 100 µM diethylcarbamazine significantly increased the hyperpolarization produced by 1 µM emodepside to −10.0±2.0 mV (p<0.01, n = 15 *unpaired t-test*, Fig. 3B). The hyperpolarization caused by 1 µM emodepside plus 100 µM diethylcarbamazine was sustained even after washing as are the effects of emodepside alone, an effect that may be explained by the lipophilic nature of the compounds and/or accumulation of second messengers in a signaling cascade [11].

## Discussion

Diethylcarbamazine is used mostly for treatment of filariasis in humans but has been used for treatment of intestinal nematode parasites [20]. Details of its mode of action remain to be defined however; it has been suggested that the effects of diethylcarbamazine are mediated via the host innate immune system [15] rather than by a direct effect on the parasite. Our observations show that 100 µM (but not 10 µM) diethylcarbamazine has a direct effect on the parasite (independent of the host) raising the possibility that its therapeutic mode of action also involves a direct effect. The antifilarial action of diethylcarbamazine appears to involve host arachidonic acid metabolism via cyclooxygenase & 5-lipoxygenase and, in addition nitric oxide metabolic pathways via inducible nitric oxide synthase [16]. A role for nitric oxide and the inducible nitric oxide pathway is suggested by the experiments involving microfliarial infected iNOS^−/−^ mice which showed no clearance response following treatment with diethylcarbamazine [16].

In addition to the treatment of filariasis, diethylcarbamazine, as a single dose treatment, has modest effects on intestinal nematode parasite infections including ascariasis and trichuriasis but is more effective when combined with ivermectin or albendazole [21]. Although diethylcarbamazine is a piperazine derivative, diethylcarbamazine does not mimic the effects of piperazine by acting as a GABA agonist on parasite muscles [22]. Here we observed that diethylcarbamazine, in the presence of sufficient calcium, had a direct effect on the worm preparation and increased activation of the SLO-1 K^+^ channel currents by shifting the *V_50_* in the hyperpolarizing direction. SLO-1 channels are calcium-dependent K^+^ channels that are pharmacologically different from delayed rectifier K^+^ channels. In low-calcium, even high concentrations of diethylcarbamazine (mM) do not activate SLO-1 K^+^ currents showing that this action requires calcium [22]. High, mM concentrations of diethylcarbamazine, in low-calcium conditions, inhibits nicotinic acetylcholine currents and a delayed rectifier K^+^ current [22] but these high-concentration effects are non-selective.

The effect of diethylcarbamazine here in our parasite preparations is to produce opening of SLO-1 K^+^ channels that does not involve the host. The outcome will be a reduction in the excitability of nerve and muscles tissues where the channels are present. A reduction in muscle excitability is predicted to inhibit motility and thus lead to exclusion from the intestinal tract. The *Ascaris* SLO-1 K^+^ channels, which are activated by calcium, are also activated by nitric oxide and emodepside [10], [11], [19]. Vertebrate SLO-1 K^+^ (BK) channels are activated by calcium, nitric oxide, arachidonic acid and fatty acid metabolites [23], [24] but are less sensitive to emodepside [25]. The synergistic action of diethylcarbamazine and emodepside on membrane potential and the voltage-activated SLO-1 K^+^ current seen in our *Ascaris* experiments could be explained by a combination of effects on SLO-1 K^+^ channels by: a) effects of diethylcarbamazine on *Ascaris* arachidonic metabolic pathways; b) effects of diethylcarbamazine on *Ascaris* nitric oxide pathways and; c) direct effects of emodepside on nematode SLO-1 K^+^ channels [10], [25].

In conclusion, our observations show that diethylcarbamazine has a direct effect on a nematode parasite and its effects synergize with emodepside on SLO-1 K^+^ channels. These observations suggest the potential for the combination of diethylcarbamazine and emodepside as an anthelmintic treatment.

## References

[1] Hotez PJ, Brindley PJ, Bethony JM, King CH, Pearce EJ, et al (2008) Helminth infections: the great neglected tropical diseases. The Journal of Clinical Investigation 118: 1311–1321. 18382743 10.1172/JCI34261 PMC2276811

[2] Kotze AC, Hunt PW, Skuce P, von Samson-Himmelstjerna G, Martin RJ, et al (2014) Recent advances in candidate-gene and whole-genome approaches to the discovery of anthelminthic resistance markers and the description of drug/receptor interactions. Int J. Parasitol: Drugs and Drug Resistance http://dx.doi.org/10.1016/j.ijpddr.2014.07.007 4: 164–184.. 10.1016/j.ijpddr.2014.07.007 PMC4266812 25516826

[3] Diawara L, Traore MO, Badjii A, Bissan Y, Doumbia Y, et al.. (2009) Faesibility of Onchocerciasis Elimination with Ivermectin Treatment in Endemic Foci in Africa: First evidence from Studies in Mali and Senegal. PLoS Neglected Tropical Diseases. DOI: 10.137/journal.pntd.0000497 3: e497 10.1371/journal.pntd.0000497 PMC2710500 19621091

[4] Nana-Djeunga HC, Bourguinat C, Pion SD, Kamgno J, Nijiokou F, et al (2012) Single nucleotide polymorphisms in β-tubulin selected in *Onchocerca volvulus* following repeated ivermectin treatment: possible indication of resistance selection. Mol Biochem Parasitol 185(1): 10–18. 22677339 10.1016/j.molbiopara.2012.05.005

[5] Craig T (2006) Anthelmintic resistance and alternative control methods. Vet Clin North Am Food Anim Pract 22(3): 567–581. 17071353 10.1016/j.cvfa.2006.07.003

[6] Hu Y, Platzer E, Bellier A, Aroian R (2010) Discovery of a highly synergistic anthelmintic combination that shows mutual hypersusceptibility. Proc Natl Acad Sci USA 107(13): 5955–5960. 20231450 10.1073/pnas.0912327107 PMC2851895

[7] Harder A, Holden-Dye L, Walker R, Wunderlich F (2005) Mechanisms of action of emodepside. Parasitol Res 97: S1–S10. 16228263 10.1007/s00436-005-1438-z

[8] Holden-Dye L, Crisford A, Welz C, von Samson-Himmelstjerna G, Walker R, et al (2012) Worms take to the slo lane: a perspective on the mode of action of emodepside. Invert Neurosci 12(1): 29–36. 22539031 10.1007/s10158-012-0133-x PMC3360863

[9] Krucken J, Harder A, Jeschke P, Holden-Dye L, O'Connor V, et al (2012) Anthelmintic cyclooctadepsipeptides: complex in structure and mode of action. Trends Parasitol 28(9): 385–394. 22858281 10.1016/j.pt.2012.06.005

[10] Martin RJ, Buxton S, Neveu C, Charvet C, Robertson AP (2012) Emodepside and SLO-1 potassium channels: A review. Exp Parasitol 132(1): 40–46. 21910990 10.1016/j.exppara.2011.08.012 PMC3262936

[11] Buxton S, Neveu C, Charvet C, Robertson AP, Martin RJ (2011) On the mode of action of emodepside: slow effects on membrane potential and voltage-activated currents in *Ascaris suum* . Brit J Pharmacol 164: 453–470. 21486286 10.1111/j.1476-5381.2011.01428.x PMC3188918

[12] Hudson A, Nwaka S (2007) The concept paper on the helminth drug initiative. Onchocerciasis/lymphatic filariasis and schistosomiasis: opportunities and challenges for the discovery of new drugs/diagnostics. Expert Opinion Drug Discovery 2: S3–7. 10.1517/17460441.2.S1.S3 23489030

[13] Townson S, Freeman A, Harris A, Harder A (2005) Activity of the cyclooctadepsipeptide emodepside against *Onchocerca gutturosa, Onchocerca lienalis* and *Brugia pahangi* Am J Trop Med Hyg 73 (Suppl 6).

[14] Zahner H, Taubert A, Harder A, Von Samson-Himmelstjerna G (2001) Effect of Bay 44-4400, a new cyclodepsipeptide, on developing stages of filariae (*Acanthocheilonema viteae, Brugia malayi, Litomosoides sigmodontis*) in the rodent *Mastomys coucha* . Acta Tropica 80: 19–28. 11495640 10.1016/s0001-706x(01)00144-9

[15] Maizels R, Denham D (1992) Diethylcarbamazine (DEC): immunopharmacological interactions of an anti-filarial drug. Parasitology 105 Suppl: S49–60. 1308929 10.1017/s0031182000075351

[16] McGarry HF, Plant LD, Taylor MJ (2005) Diethylcarbamazine activity against *Brugia malayi* microfilariae is dependent on inducible nitric-oxide synthase and the cyclooxygenase pathway. Filaria Journal 4: 4. 15932636 10.1186/1475-2883-4-4 PMC1173132

[17] Bolotina V, Najibi S, Palacino J, Pagano P, Cohen R (1994) Nitric oxide directly activated calcium-dependent potassium channels in vascular smooth muscle. Nature 368 (6474): 850–853. 10.1038/368850a0 7512692

[18] Bascal ZA, Cunningham JM, Holden-Dye L, O'Shea M, Walker RJ (2001) Characterization of a putative nitric oxide synthase in the neuromuscular system of the parasitic nematode, *Ascaris suum* . Parasitol 122: 219–231. 10.1017/s003118200100720x 11272653

[19] Bowman JW, Winterrowd CA, Friedman AR, Thompson DP, Klein RD, et al (1995) Nitric oxide mediates the inhibitory effects of the SDPNFLRFamide, nematode FMFRamide-related neuropeptide, in *Ascaris suum* . J Neurophys 74: 1880–1888. 10.1152/jn.1995.74.5.1880 8592181

[20] Ghenem MH (1954) The treatment of Ascariasis and Ancylostomiasis with Hetrazan (diethylcarbamazine). Trans. Roy. Soc. Trop. Med. Hygiene 48: 73–76. 10.1016/0035-9203(54)90040-1 13136414

[21] Belizario VY, Amarillo ME, De Leon WU, De los Reyes AE, Bugayong MG, et al (2003) A comparison of the efficacy of single doses of albendazole, ivermectin, and diethylcarbamazine alone or in combinations against *Ascaris* and *Trichuris spp* . Bull World Health Organ: Int J Public Health 81(1): 35–42. PMC2572315 12640474

[22] Martin RJ (1982) Electrophysiological effects of piperazine and diethylcarbamazine on *Ascaris suum* somatic muscle. Brit J Pharmacol 77: 255–265. 7139188 10.1111/j.1476-5381.1982.tb09294.x PMC2044588

[23] Ahn D, Kim Y, Lee Y, Kang B, Kang D (1994) Fatty acids directly increase the activity of Ca2+-activated K+ channels in rabbit coronary smooth muscle cells. Yonsei Med J 35(1): 10–24. 8009892 10.3349/ymj.1994.35.1.10

[24] Meves H (1994) Modulation of ion channels by arachidonic acid. Prog Neurobiol 43(2): 175–186. 7526418 10.1016/0301-0082(94)90012-4

[25] Crisford A, Murray C, O'Connor V, Edwards R, Kruger N, et al (2011) Selective toxicity of the anthelmintic emodepside revealed by heterologous expression of human KCNMA1 in *Caenorhabditis elegans* . Mol Pharmacol 79(6): 1031–1043. 21415309 10.1124/mol.111.071043 PMC3102553

